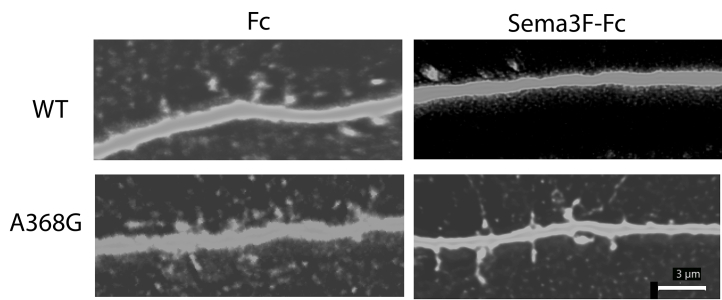# Structural interactions of ankyrin B with NrCAM and β2 spectrin

**DOI:** 10.1016/j.jbc.2025.110872

**Published:** 2025-10-30

**Authors:** Venkat R. Chirasani, Victoria A. Haberman, Erik N. Oldre, Barrett D. Webb, Ernest B. Pereira, Wonsuk Yang, Patricia F. Maness

**Affiliations:** 1 Department of Biochemistry and Biophysics, University of North Carolina School of Medicine at Chapel Hill, Chapel Hill, North Carolina, USA; 2 R. L. Juliano Structural Bioinformatics Core, University of North Carolina School of Medicine at Chapel Hill, Chapel Hill, North Carolina, USA

**Keywords:** ankyrin, autism spectrum disorder, molecular modeling, cell adhesion molecules, spectrin

## Abstract

Ankyrin 2 is a high confidence autism spectrum disorder (ASD) gene encoding the spectrin-actin scaffold protein Ankyrin B (AnkB). The 220 kDa isoform of AnkB has multiple functions including developmental spine pruning through L1 family cell adhesion molecules (L1-CAMs) and class 3 Semaphorins on dendrites of pyramidal neurons to achieve an appropriate excitatory balance in the neocortex. Molecular modeling employing AlphaFold was used to predict the structure and interactions of AnkB with the cytoplasmic domain of neuron-glial related L1-CAM (NrCAM), and with β2-Spectrin. The validity of the models was assessed by analyzing protein-protein interactions by co-immunoprecipitation from HEK293 cell lysates after mutating key residues in AnkB predicted to impair these associations. Results revealed a pocket with critical residues in the AnkB membrane-binding domain that engages NrCAM at the conserved cytoplasmic motif – FIGQY. AlphaFold modeling of the AnkB/β2-Spectrin complex identified key interactions between the AnkB spectrin-binding domain and β2-Spectrin repeats 14 to 15. Selected ASD-linked mutations in AnkB predicted to impact binding to NrCAM or β2-Spectrin were then assayed for protein interactions. Maternally inherited ASD missense mutations AnkB A368G located in the NrCAM binding pocket and AnkB R977Q in the Zu5A subdomain disrupted associations with NrCAM and β2-Spectrin, respectively. Moreover, AnkB A368G impaired the neuronal function of 220 kDal AnkB for Semaphorin 3F-induced spine pruning in mouse cortical neuron cultures. These new findings provide structural insights into the L1-CAM/AnkB complex and the molecular basis of ASD etiology associated with AnkB missense mutations.

Large scale genomic studies have revealed a diversity of genetic mutations that increase disease risk in autism spectrum disorder (ASD) and other neurodevelopmental diseases (1, 2, 3). Many ASD risk genes are linked to altered development of pyramidal neurons (4, 5), principal neurons whose dendritic spines harbor the vast majority of excitatory synapses in the neocortex. Cortical hyper-expansion and brain enlargement are vulnerabilities for ASD that become apparent in childhood at the end of year 1 (6, 7, 8). Abnormal social and cognitive functions characteristic of ASD are not present at this time, but arise after relevant neural circuits are formed and synaptic connections are refined (9). During normal development dendritic spines and excitatory synapses are initially overproduced, eliminated in substantial numbers in juveniles, and stabilized in adulthood (10, 11, 12, 13). In ASD and Fragile X Syndrome, elevated spine density is evident in the adult prefrontal cortex (PFC) (14, 15, 16, 17, 18), where essential circuits contribute to social and cognitive function (19). Defective pruning of overproduced spines is therefore a cogent hypothesis for increased spine density and certain behavioral deficits associated with ASD.

We identified a molecular mechanism during postnatal development of the mouse prefrontal cortex, in which class 3 Semaphorins (Sema3s) drive activity-dependent spine elimination on pyramidal neurons (20). Sema3s bind receptor complexes comprising family cell adhesion molecule (L1-CAMs) and their coreceptors Neuropilin-1/2 and PlexinA1-4 (PlexA1-4) (21, 22, 23, 24) (Fig. 1*A*). Neuron-glial related L1-CAM (NrCAM) is an L1 family member that together with Npn-2 and PlexA3 forms a holoreceptor for Sema3F. Another L1-CAM Close Homolog of L1 (CHL1) forms a holoreceptor with Npn-2 and PlexA4 for Sema3B (22, 23). This specificity allows Sema3F to prune spine subpopulations through NrCAM, while Sema3B prunes distinct spine subpopulations through CHL1. L1, the prototype of the L1-CAM family, binds Npn-1, a coreceptor for Sema3A (25) and also regulates spine density (26). Selective spine pruning through L1-CAMs occurs only on apical, not basal, dendrites of cortical pyramidal neurons. Apical and basal dendrites are functionally specialized for integrating synaptic inputs and propagating electrical signals (27).

**Figure 1:**
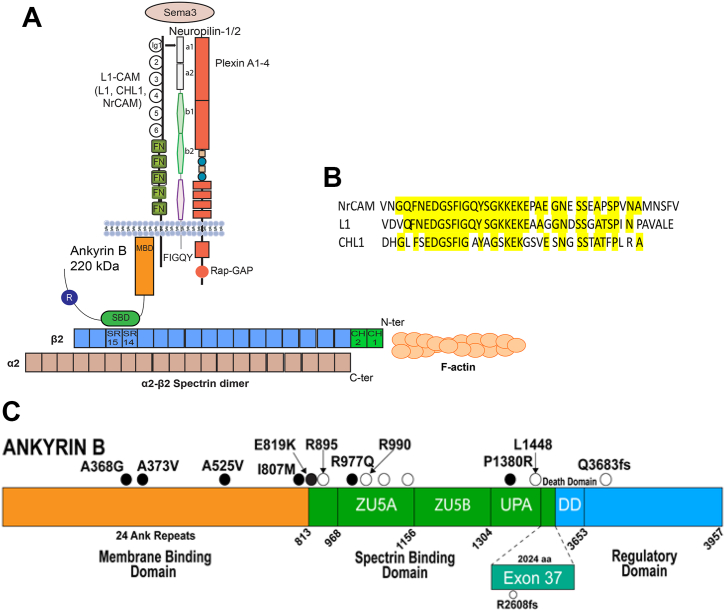
**Semaphorin 3 Holoreceptor Complex with Conserved L1-CAM Cytoplasmic Domains, and AnkB Domain Structure with Sites of Human ASD Mutations.***A*, scheme of secreted class 3 Semaphorins (Sema3) bound to holoreceptor complex of L1 cell adhesion molecules (L1-CAMs) (L1, CHL1, neuron-glial related cell adhesion molecule (NrCAM)), Neuropilins-1/2, and PlexinA1-4. The Ig1 domain of L1-CAMs binds to the a1 domain of Neuropilin-1/2. Downstream signaling through the intrinsic Rap-GAP activity of PlexinA culminates in dendritic spine pruning in neurons. L1-CAM cytoplasmic domain binds ankyrin B (AnkB) (220 kDa, shown) at its membrane-binding domain (MBD) which contains the motif FIGQY. AnkB also harbors a C-terminal death and regulatory domain.The AnkB spectrin-binding domain (SBD) binds β2 Spectrin at spectrin repeats SR14 and SR15. Shown is a heterodimer of α2-β2 Spectrin, which predominates in brain. β2-Spectrin consists of 17 spectrin repeats (SR1-17) and two N-terminal calponin homology domains (CH1,CH2), which bind F-actin. α2-Spectrin consists of 20 SRs with SR19-SR20 comprising the heterodimerization domain. Spectrin heterodimers assemble into heterotetramers containing two α and two β subunits. The relative sizes of the proteins are approximated in the linear domain structures shown. *B*, sequences of the conserved cytoplasmic domains of NrCAM, L1, and CHL1. Alignment shows identical residues in *yellow*, including the Ankyrin binding motif FIGQ/AY and flanking residues. *C*, AnkB-220 domains with sites of human autism spectrum disorder (ASD) mutations: ● missense; ○ nonsense, frameshift and deletion (fs). The AnkB MBD contains 24 ANK repeats. The SBD includes ZU5A/B and UPA subdomains. The death domain and regulatory domain are located in the C-terminal region. A large (2024 amino acid) insert encoded by exon 37 of the *Ank2*, Ankyrin 2 (*Ank2*) gene generates the AnkB-440 splice variant. AnkB, ankyrin B; L1-CAM, L1 cell adhesion molecule; MBD, membrane-binding domain; NrCAM, neuron-glial related cell adhesion molecule; SBD, spectrin-binding domain; Sema3, 3 semaphorins.

All L1-CAMs bind Ankyrin B (AnkB), a spectrin-actin adapter protein that is encoded by the high confidence ASD risk gene Ankyrin 2 (*Ank2*) (category 1, SFARI Gene Database) (Fig. 1, *A* and *C*). There are two principal AnkB spliced isoforms in the brain (220- and 440-kDa) (28). AnkB-220 is localized to the somatodendritic compartment of neurons (29) and mediates Sema3-induced spine pruning (30), whereas AnkB-440, which contains a large insert encoded by exon 37, is axonal and suppresses axon branching (31, 32) (Fig. 1*C*). L1-CAMs engage Ankyrins through the highly conserved L1-CAM cytoplasmic domain, which includes the motif FIGQY (FIGAY in CHL1) (Fig. 1*B*) (33, 34, 35). Mutation of FIGQY to FIGQH in L1 is associated with a human intellectual disability syndrome with varying degrees of hydrocephalus, aphasia, and spastic paraplegia (36, 37). L1-FIGQH knock-in mouse models are defective in AnkB binding and exhibit increased spine density on apical dendrites (26), axon guidance errors (38), and unstable interneuron synapses onto pyramidal cells (39, 40). Global L1 KO mice also display increased spine density (26), enlarged brain ventricles, and aberrant axon guidance (41, 42, 43).

L1-CAMs bind to the Ankyrin N-terminal membrane-binding domain (MBD), which comprises 24 ANK repeats that form a superhelical solenoid structure (44) (Fig. 1*C*). The Ankyrin MBD also binds voltage-gated ion channels including Nav1.2 (29). Binding sites for Nav1.2 and the L1-CAM Neurofascin (160 kD) are distributed across ANK repeats and partially overlap but have not been fully defined (44). Spectrins are Actin-binding proteins comprising elongated heterotetramers of 2 α and 2 β subunits (45). The spectrin-binding domain (SBD) of Ankyrin (Zu5A, ZU5B, UPA) engages β 2-Spectrin (Fig. 1*C*), linking Ankyrin-receptor complexes to the Actin cytoskeleton (46). The C-terminal region of AnkB contains a death domain and regulatory domain (Fig. 1*C*). Human *de novo* and inherited ASD variants have been reported across the *Ank2* sequence of both principal isoforms of AnkB (Fig. 1*C*) (2, 47, 48, 49, 50, 51, 52). These variants include missense and truncating mutations in the AnkB MBD and SBD, which are common to both isoforms. Genetic variants in β2-Spectrin are also associated with autism-like neurodevelopmental syndromes (45, 53, 54). The molecular interactions between the L1-CAM cytoplasmic domain and AnkB MBD, and between β-Spectrin and the AnkB SBD have not been defined in detail, in part due to the challenge of analyzing their extended protein architectures.

Here we combined molecular modeling using AlphaFold with a biochemical approach to predict and identify the structural interactions of AnkB with the conserved cytoplasmic domain of NrCAM and with β2-Spectrin. We generated selected mutations in the AnkB MBD and SBD and assessed their impact on binding to NrCAM or β2-Spectrin by co-immunoprecipitation. Results revealed a pocket in ANK repeat R11 of the AnkB MBD that targets the NrCAM FIGQY motif. A maternally inherited ASD missense mutation in AnkB (A368G) located in this pocket disrupted association with NrCAM and functionally impaired Sema3F-induced spine pruning in cortical neurons. The AnkB/β2-Spectrin model identified key interactions between the AnkB SBD and spectrin repeats 14 and 15. A maternally inherited ASD missense mutation (R977Q) in the Zu5A subdomain of the AnkB SBD disrupted association with β2-Spectrin. These new findings provide insight into the L1-CAM/AnkB complex and molecular basis of ASD etiology associated with AnkB missense mutations.

## Results

### Structural modeling and experimental validation of the AnkB-NrCAM complex

Our initial goal was to predict key amino acids mediating binding between AnkB and the L1-CAM cytoplasmic domain, and then to assess whether ASD variants in the predicted AnkB region might alter the interactions. The lack of experimentally resolved structures or appropriate templates for homology modeling posed significant challenges for accurate structure prediction. To address this, we employed the deep learning-based protein structure prediction tool AlphaFold v2.2. We focused on NrCAM due to its role in Sema3F-induced spine pruning mediated by AnkB-220 (30). The cytoplasmic domain of NrCAM (residues 1191–1304, Uniprot ID: Q92823) was modeled in association with ANK repeats 1 to 24 of the MBD (residues 30–822, Uniprot ID: Q01484). We generated five models for the AnkB-NrCAM complex and all models displayed the reported hydrophobic interface, with one out of five displaying an alternative orientation of NrCAM’s disordered region. The RMSD among five models varied from 0.868 to 1.427 Å, possibly due to the long-disordered region in NrCAM. However, certain key interactions were retained in all five models (Table S1).

**Figure 2:**
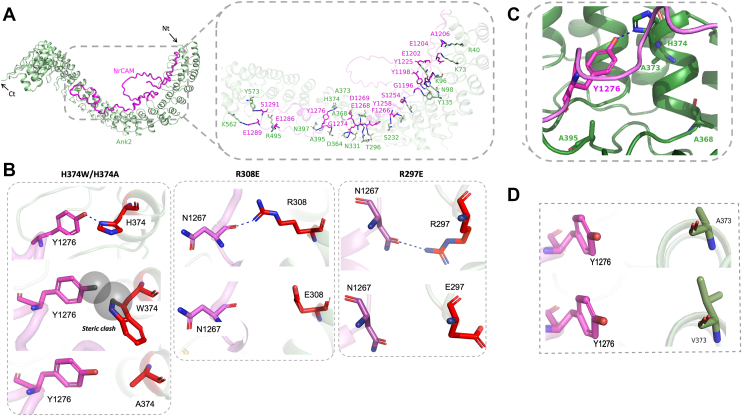
**AlphaFold Model of the AnkB Membrane-Binding Domain Bound to the NrCAM Cytoplasmic Domain.***A*, AlphaFold-predicted structure of the AnkB/NrCAM complex, showing the NrCAM cytoplasmic domain (residues 1191–1304, *magenta*) bound to the AnkB MBD, comprising helical ANK repeats 1 to 24 (*green*). *Arrows* indicate the N- and C-terminal ends (Nt, Ct). *B*, structural models generated using the PyMOL mutagenesis tool illustrate how AnkB mutations H374W, H374A, R308E, and R297E (*red*) alter interactions with NrCAM residues Y1276 and N1267 (*magenta*). H-bonding, blue dashed lines. *C*, Alphafold-predicted structure of an AnkB binding pocket (*green*) showing proximity of NrCAM FIGQY1276 (*magenta*) with AnkB H374, A373, and A368. H-bonding, *blue dashed lines*. *D*, structural model generated using the PyMol mutagenesis tool illustrating how the AnkB mutation A373V (*green*), which is in proximity to NrCAM Y1276 (*magenta*), may increase binding to NrCAM due to the bulkier side chain of valine compared to alanine. AnkB, ankyrin B; MBD, membrane-binding domain; NrCAM, neuron-glial related cell adhesion molecule.

**Supporting Figure S1:**
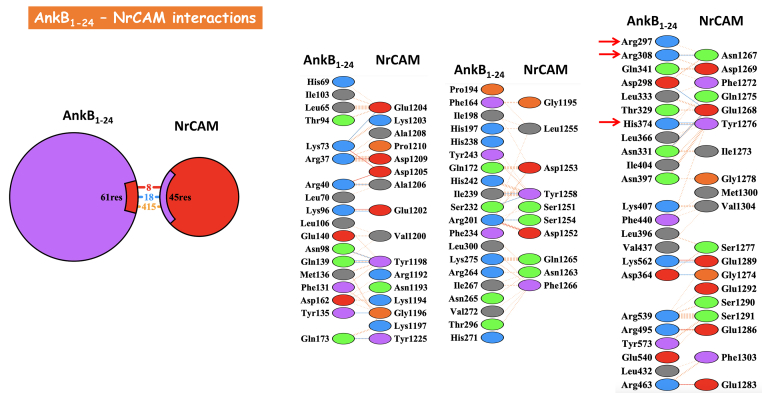


The AlphaFold model of the NrCAM-AnkB complex revealed critical interactions between the cytoplasmic domain of NrCAM and AnkB (Fig. 2*A*). A disordered loop in the NrCAM sequence of about 40 amino acids protruded from the helical ANK repeats of AnkB. Interaction analysis by PDBsum identified eight salt bridges,18 H-bonds, and 415 non-bonded contacts between AnkB and NrCAM (Fig. S1). The NrCAM interaction site in AnkB contained a pocket comprising H374 and two residues A368 (55) and A373 (47) that are sites of genetic variation in ASD (Fig. 2, *B*–*D*). In the model Y1276 located in the FIGQY motif of NrCAM formed a strong hydrogen bond with H374 in the ANK R11 repeat, potentially stabilizing the complex (Figs. 2, *B*, *C* and S1). In addition to H-bonds, hydrophobic contacts were identified between surrounding residues, reinforcing the interaction interface. PDBsum analysis revealed a hydrophobic core involving AnkB residues R297, R308, and H374, which form non-bonded interactions with NrCAM’s cytoplasmic domain (Fig. S1 arrows). Specifically, R297 interacts with N1267, R308 with N1267 and D1269, and H374 with F1272, Q1275, and Y1276, primarily through hydrophobic contacts. Table S2 lists characteristics of interacting pairs including types (*e.g.*, salt bridges, H-bonds) and bond distances.

**Supporting Figure S2:**
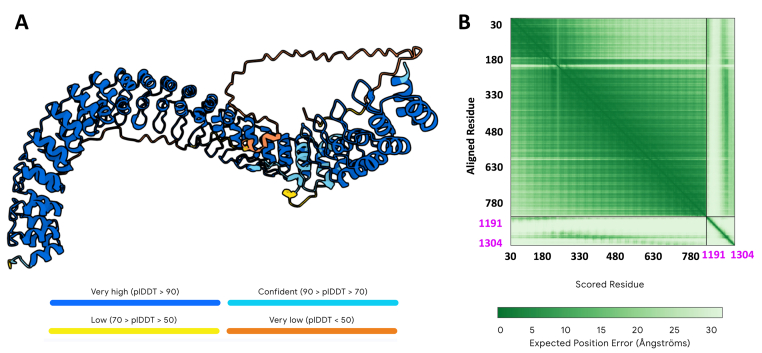


The structural model yielded a predicted TM-score (pTM) of 0.73 and inter-chain predicted TM score (ipTM) of 0.42, indicating moderate confidence in the predicted interface. To address which regions of the structural models had reduced confidence scores, the NrCAM/AnkB model was further analyzed for its per-residue confidence scores, specifically by the Local Distance Difference Test (pLDDT). The pLDDT, a per-atom estimate of AlphaFold's confidence in a structure prediction (0–100, higher is better), revealed that the cytoplasmic domain of NrCAM (residues 1191–1304) ad notably low pLDDT scores (<50) (Fig. S2). The low scores suggested high structural variability and wereindicative of regions where the prediction was unreliable. Conversely, the core AnkB repeats (residues 30–822) showed higher confidence, with pLDDT scores exceeding 70, reflecting a more reliable structural prediction for these regions.

**Figure 3:**
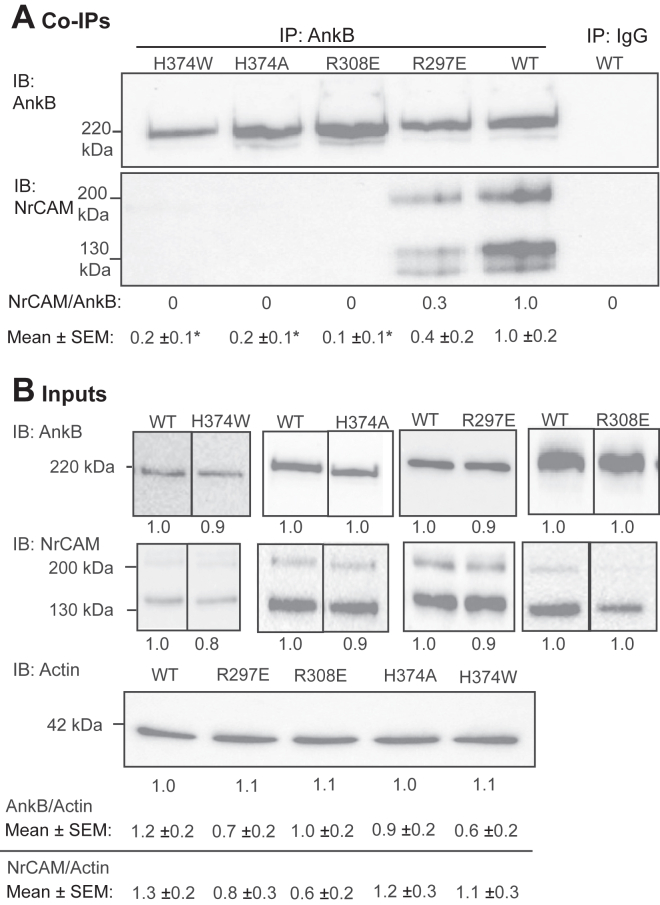
**AnkB Associates with NrCAM at a Nexus of Residues in ANK Repeat R11.***A*. NrCAM co-immunoprecipitates with WT AnkB-220 (HA-tagged) from transfected HEK293 cells, and to a much lesser extent with HA-tagged AnkB-220 bearing point mutations H374W, H374A, R308E, and R297E. AnkB was immunoprecipitated from cell lysates with anti-HA antibodies, and immunoblotting (IB) with anti-NrCAM antibodies followed by reprobing with anti-HA antibodies. NrCAM/AnkB ratios in the immunoprecipitates relative to WT are shown under each lane of a representative blot. Mean ratios of NrCAM/AnkB ± SEM from five replicate experiments for each WT or mutant are indicated below. Significant mean differences (2-tailed *t* test, *p* < 0.05) between WT and mutant AnkB were: ∗H374W (*p* = 0.002), ∗H374A (*p* = 0.003), ∗R308E (*p* < 0.001), and ∗ R297E (*p* = 0.03). Nonimmune IgG did not immunoprecipitate AnkB or NrCAM. *B*. Input WT and mutant HA-AnkB-220 levels in HEK293 lysates (5 μg) prior to immunoprecipitation were determined by IB with anti-HA antibodies, followed by stripping and reprobing with anti-NrCAM antibodies, and then with anti-Actin antibodies as loading control. Mutant values relative to WT in each representative blot are shown below the lanes. Lanes from the same blot are shown without a line, or with a line if lanes were not adjacent. Blots from different gels are shown with a wider separation. Mean ratios of AnkB/Actin or NrCAM/Actin ± SEM from n replicate experiments are indicated below. There was not a significant mean difference in AnkB/Actin (2-tailed *t* test, *p* > 0.05) between WT AnkB (n = 12) and H374W (n = 5, *p* = 0.08), H374A (n = 6, *p* = 0.31), R297E (n = 5, *p* = 0.13), R308E (n = 6, *p* = 0.08). There was also not a significant mean difference in NrCAM/Actin between WT and H374W (*p* = 0.39), H374A (*p* = 0.54), R297E (*p* = 0.09), and R308E (*p* = 0.06). The position of the 220 kDa molecular weight marker from PageRuler Plus is indicated and coincides with the AnkB220-HA band. AnkB, ankyrin B; IB, immunoblotting; NrCAM, neuron-glial related cell adhesion molecule.

**Supporting Figure S3:**
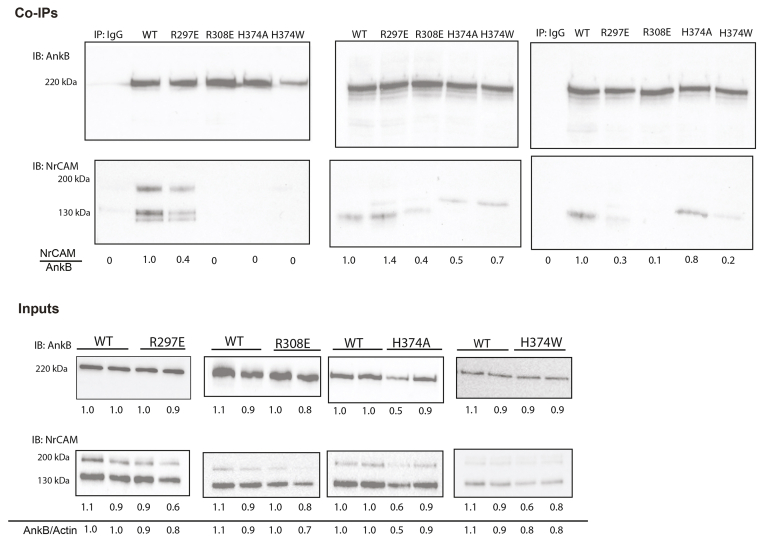


To ensure the robust interpretation of experimental results, AnkB mutations (*e.g.*, H374W, H374A, R308E, R297E) were strategically introduced into the high-confidence regions of the interface, which could disrupt both hydrophilic and hydrophobic interactions with NrCAM, and assayed by co-immunoprecipitation. Mutations were generated in the 220 kD isoform of AnkB by site-directed mutagenesis of the cDNA sequence. Because AnkB-220, not AnkB-440, mediates Sema3F-Fc induced spine pruning in neuronal cultures (30), mutations were engineered into AnkB-220 cDNA with a C-terminal 2X HA-tag. Mutations were confirmed by whole plasmid DNA sequencing. Expression plasmids encoding WT AnkB-220-HA and mutant AnkB-220-HA proteins were co-transfected with plasmids encoding WT NrCAM cDNA in equimolar amounts into HEK293 cells and assayed for association by co-immunoprecipitation from cell lysates prepared in the nonionic detergent Brij98. AnkB-HA was immunoprecipitated with anti-HA antibodies adsorbed to Protein A/G magnetic beads. Immune complexes were separated by SDS-PAGE and transferred to nitrocellulose. Western blotting was carried out using NrCAM antibodies, followed by reprobing for AnkB with anti-HA antibodies. Co-immunoprecipitation (co-IP) from transfected HEK293 cells showed that NrCAM effectively co-IP with WT AnkB, as seen from associated NrCAM proteins of 220 kDa and ∼130 kDa (Fig. 3*A*). Nonimmune IgG did not pull down AnkB or NrCAM (Fig. 3*A*). We compared co-immunoprecipitation of NrCAM with WT AnkB *versus* AnkB containing Trp or Ala substitutions at the H374 residue, as these mutations were predicted to perturb the interaction. Relative levels of NrCAM (220- and ∼130 kDa combined) and AnkB-HA were quantified by densitometric scanning of labeled bands (Fig. 3*A*). AnkB H374W and H374A were found to be strongly inhibited for NrCAM association. Quantification of blots from multiple replicate experiments (Fig. S3) yielded mean NrCAM/AnkB ratios (±SEM) that were significantly decreased for both AnkB mutants relative to WT (Fig. 3*A*; two-tailed *t* test, ∗*p* < 0.05). NrCAM and other L1-CAMs (200 kDa) are proteolytically processed to N-terminal 130 to 150 kDa and 60 kDa C-terminal fragments (56, 57). The fragments remain attached to the cell by homophilic and heterophilic binding interactions (58, 59), although some may be released. They are the predominant species in mouse brain and vary in abundance depending on cell type, environment, and signaling (60). NrCAM antibodies to the extracellular domain used here recognized only the 220 kDa and ∼130 kDa proteins.

To better understand their impact on the complexes, the PyMOL mutagenesis tool was employed to model the mutations. Substitution of H374 with tryptophan in the AnkB structural model introduced steric clashes with Y1276 in the FIGQY motif due to the larger side chain of tryptophan, disrupting the H-bond and causing loss of structural integrity within the AnkB-NrCAM complex (Fig. 2*B*). Furthermore, replacing H374 with alanine prevented formation of the crucial H-bond with Y1276, reducing the stability of the complex and weakening the overall AnkB-NrCAM interface (Fig. 2*B*).

Computational mutagenesis showed that AnkB R308 promotes stability of the AnkB/NrCAM complex by forming a stabilizing H-bond with N1267 in the NrCAM cytoplasmic domain sequence QFN^1267^EDGSFIGQY^1276^(Fig. 2*B*). A charge substitution replacing R308 with glutamate (R308E) could prevent this interaction, destabilizing the local conformation. We generated the R308E substitution in AnkB-220 and assayed its association with NrCAM by co-immunoprecipitation from transfected HEK293 cells. This mutation resulted in nearly a complete loss of binding to NrCAM (Figs. 3*A* and S3). Additionally, N1267 in NrCAM can form a stabilizing H-bond with R297 in AnkB (Fig. 2*B*). Substitution of Glu for Arg at position 297 should disrupt this interaction. Accordingly, the charge substitution R297E in AnkB was found to decrease co-immunoprecipitation with NrCAM by ∼60% (Figs. 3*A* and S3). Overall, the association studies showed that the mutations reduced binding affinity by 60% (R297E), 90% (R308E), and 80% (H374W, H374A) compared to WT, confirming a vital role of hydrophobic and hydrophilic interactions in complex stability.

To evaluate whether the AnkB mutant proteins were stably expressed and not degraded, HEK293 cells were transfected with WT or mutant AnkB cDNAs, together with NrCAM cDNA in equimolar quantities. Equal amounts of cell lysate protein were analyzed by Western blotting. AnkB and NrCAM protein bands were quantified by densitometric scanning and normalized to corresponding WT levels. As shown in representative input blots, AnkB mutants H374W, H374A, R297E, and R308E were expressed at equivalent levels to WT, as was NrCAM (Fig. 3*B*). Blots were reprobed for endogenous Actin as a loading control. Evaluating AnkB/Actin and NrCAM/Actin ratios, all AnkB mutants and NrCAM were expressed at levels comparable to WT. Replicate experiments yielded similar results (Fig. S3). Mean AnkB/Actin and NrCAM/Actin ratios from replicate assays are presented below the blots.

### ASD-related variants in AnkB are impaired for NrCAM binding

**Figure 4:**
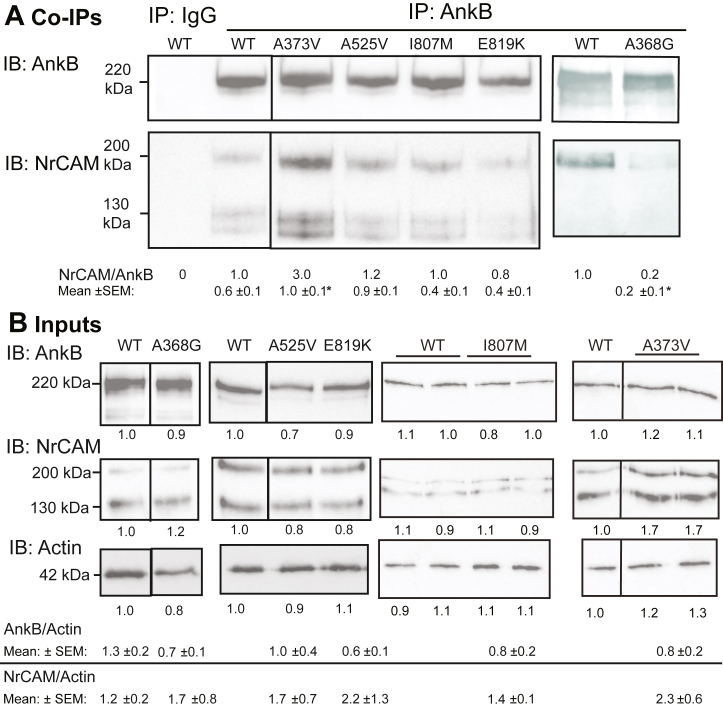
**Altered Interactions of Missense ASD Mutations in AnkB A368G and A373V with NrCAM.***A*. NrCAM co-immunoprecipitated with HA-tagged WT AnkB-220 from transfected HEK293 cells with anti-HA antibodies but not nonimmune IgG. ASD mutation A373V showed enhanced association with NrCAM; A368G mutation showed decreased association with NrCAM. NrCAM/AnkB ratios were obtained by densitometry as presented under the representative blots. Lanes from the same blot are shown without a line or with a line if not adjacent. Blots from different gels are shown with a wider separation. Mean ratios of NrCAM/AnkB ± SEM from n replicate experiments for WT and mutants are indicated below. Mean differences (2-tailed *t* test, ∗*p* < 0.05) were significant between WT and mutant AnkB for ∗A373V (n = 3, *p* = 0.02) and ∗ A525V (n = 3, *p* = 0.15), but not for I807M (n = 5, *p* = 0.15) or E819K (n = 3, *p* = 0.23). *B*. Input WT and mutant HA-AnkB-220 levels in HEK293 lysates (5 μg) prior to immunoprecipitation were determined by IB with anti-HA antibodies, followed by stripping and reprobing with anti-NrCAM antibodies, then with anti-Actin antibodies as loading control. Lanes from the same blot are shown without a line or with a line if not adjacent. Blots from different gels are shown with a wider separation. Levels of AnkB, NrCAM, and Actin relative to WT are shown below representative blots. Mean ratios of AnkB/Actin ± SEM from n replicate experiments indicated that there was not a significant difference in AnkB/Actin (2-tailed *t* test, *p* < 0.05) between WT (n = 10) and mutant AnkB for A368G (n = 3, *p* = 0.35), A525V (n = 3, *p* = 0.68), E819K (n = 4, *p* = 0.27), I807M (n = 4, *p* = 0.15), or A373V (n = 4, *p* = 0.40). There was also not a significant mean difference in NrCAM/Actin between WT and A368G (*p* = 0.43), A525V (*p* = 0.77), E819K (*p* = 0.40), I807M (*p* = 0.39), or A373V (*p* = 0.43). The position of the 220 kDa molecular weight marker from PageRuler Plus is indicated and coincides with the AnkB220-HA band. AnkB, ankyrin B; IB, immunoblotting; NrCAM, neuron-glial related cell adhesion molecule.

**Supporting Figure S4:**
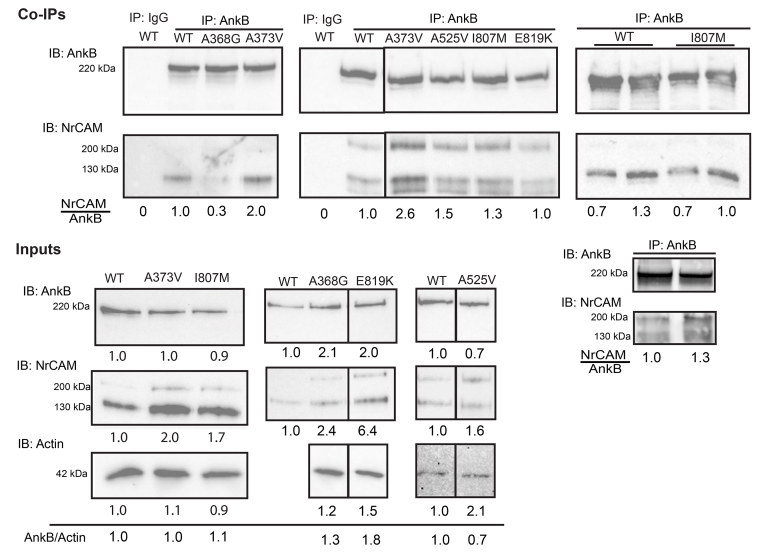


We next assessed interactions of human ASD missense variants in the AnkB MBD that were predicted by AlphaFold-v2.2 to impact NrCAM binding. We first analyzed AnkB A368G, which is a maternally inherited missense mutation in ANK repeat R11 (47, 55) The mutation was generated by site-directed mutagenesis in AnkB-220 cDNA, and analyzed for NrCAM binding by co-immunoprecipitation from transfected HEK293 cells. AnkB A368G was significantly impaired for binding to NrCAM, as evident from the decreased NrCAM/AnkB ratio compared to WT in the immunoprecipitates (Fig. 4*A*). Quantification of replicate blots (Fig. S4) yielded mean NrCAM/AnkB ratios (±SEM) that were significantly decreased for AnkB A368G compared to WT (Fig. 4*A*; two-tailed *t* test, ∗*p* < 0.05). The substitution of alanine with glycine at position 368 likely increases local conformational flexibility due to glycine’s smaller side chain and lack of β-carbon, destabilizing the binding pocket for NrCAM. Additionally, the AnkB R297-NrCAM N1267 interaction may be affected by increased flexibility in the A368G variant, further influencing optimal NrCAM binding (Fig. 2, *B* and *C*).

**Supporting Figure S5:**
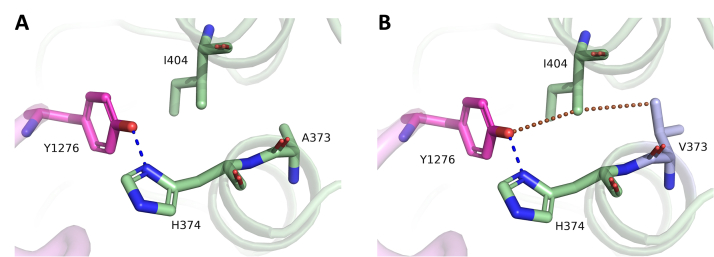


Other maternally inherited ASD mutations A373V in R11 (47), A525V in R15 (55), and E819K in R24 (55) were similarly assayed, along with the *de novo* AnkB mutation I807M in R24 (47, 61). Surprisingly, the A373V mutation, which is in proximity to Y1276 and H374, increased mean binding affinity for NrCAM by 1.7 fold compared to WT (Figs. 4*A* and S4). This might be explained by the bulkier isopropyl group of the valine side chain compared to the methyl group of the alanine side chain (Fig. 2*D*). This mutation also introduces additional hydrophobic interactions, with V373 allosterically contacting NrCAM Y1276 *via* AnkB I404 (Fig. S5). It is interesting to speculate that AnkB A373 might serve as a potential gain-of-function mutation for the AnkB/NrCAM interaction.

In contrast, AnkB variants A525V, I807M, and E819K were not significantly impacted for binding to NrCAM (Figs. 4*A* and S4). Based on PDBSum the neutral mutations A525V and I807M were not expected to alter key interactions nor introduce new ones, suggesting a minimal impact on the AnkB-NrCAM complex's overall stability. Structural modeling predicted that despite its being a charge variant AnkB E819K was also unlikely to have a binding interface with NrCAM. For these ASD mutants, replicate experiments gave similar results (Fig. S4), and quantification yielded mean NrCAM/AnkB ratios (±SEM) that were not significantly decreased relative to WT (Fig. 4*A*; two-tailed *t* test, *p* > 0.05). To confirm that the ASD-related AnkB mutant proteins were stably expressed, lysates of HEK293 cells were transfected with WT or mutant AnkB, together with equimolar amounts of NrCAM plasmid, and equal amounts of protein were analyzed by Western blotting. All of the mutants were expressed at equivalent levels to WT AnkB and showed similar AnkB/Actin ratios (Fig. 4*B*). Replicate experiments yielded similar results (Fig. S4) with mean NrCAM/AnkB ratios (±SEM) that were not significantly decreased relative to WT (two-tailed *t* test, *p* > 0.05).

### Comparison of interactions of AnkB/NrCAM with other resolved structures

**Supporting Figure S6:**
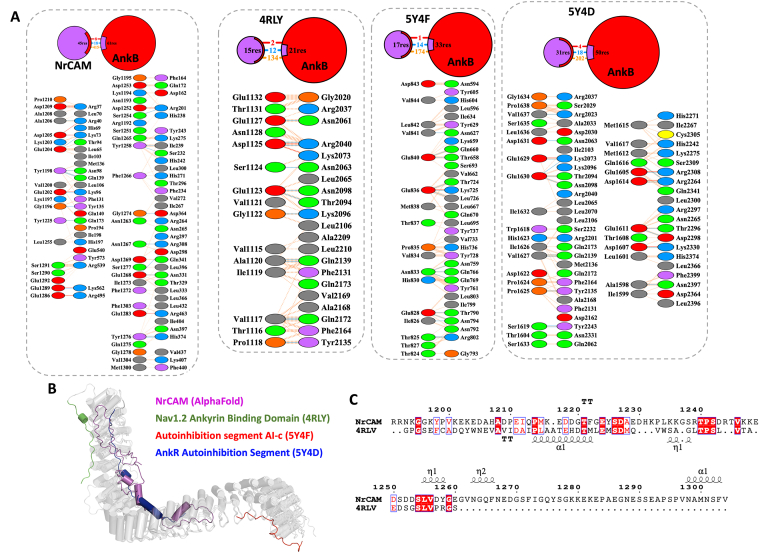


The auto-inhibitory domain of AnkB plays a critical role in modulating its interactions with binding partners. The auto-inhibiting segment of AnkB (PDB ID: 4RLV) is reported to interact with the AnkB MBD *via* a disordered region that adopts a specific binding motif upon interaction, potentially hindering access to binding sites for NrCAM and β2-Spectrin. We compared the residue interaction patterns of the AlphaFold-predicted AnkB R1-R24/NrCAM complex with experimentally resolved structures: AnkB with the AnkR autoinhibitory segment (PDB ID: 5Y4D, 5Y4F), AnkB R1-R9 with the Nav1.2 ankyrin binding domain (PDB ID: 4RLY), and AnkB R13-R24 with the autoinhibitory segment AI-c (PDB ID: 5Y4F). We used SwissModel to build missing residues/atoms in the PDB structures and analyzed interactions using PDBSum. Since 4RLV and 5Y4D are identical, we focused on 5Y4D. As seen in Figs. S1 and S6*A*, the AnkB/NrCAM model shows eight salt bridges, 18 H-bonds, and 415 non-bonded contacts, predominantly hydrophobic. Similarly, AnkB interaction with the AnkR auto-inhibitory segment (5Y4D) exhibits four salt bridges, 18 H-bonds, and 202 non-bonded contacts, with the AnkR segment binding the same NrCAM binding region of AnkB (Fig. S6*A*). In contrast, 4RLY (AnkB R1–R9 with Nav1.2 ankyrin-binding domain) has two salt bridges, 12 H-bonds, and 34 non-bonded contacts (Fig. S6*A*), which binds adjacent to the NrCAM/AnkR interface, indicating a distinct but proximal interaction site. Conversely, 5Y4F (AnkB R13–R24 with the AI-c auto-inhibitory segment) shows one salt bridge, 14 H-bonds, and 174 non-bonded contacts, binding on the opposite side of AnkB (residues 595–817), suggesting a unique interface. Structural overlays (Fig. S6*B*) confirmed that NrCAM and the autoinhibitory segment of AnkR (5Y4D) share a similar binding conformation, while Nav1.2 (4RLY) and AI-c (5Y4F) engage distinct regions, highlighting context-specific interaction patterns for AnkB.

To investigate whether NrCAM’s disordered region exhibited a similar binding motif, we performed a comparative structural analysis (Fig. S6*C*). Sequence alignment of NrCAM’s cytoplasmic domain with AnkB’s auto-inhibitory segment (4RLV) revealed limited sequence homology, suggesting distinct binding motifs. However, secondary structure predictions using PSIPRED indicated that NrCAM’s disordered region may adopt a transient helical conformation upon binding, similar to AnkB’s auto-inhibitory segment. These findings suggested that while the binding mechanisms share some similarities (*e.g.*, disorder-to-order transition), the specific motifs differ.

### Structural modeling and experimental validation of the AnkB/β2-spectrin complex and interaction with ASD variants

**Figure 5:**
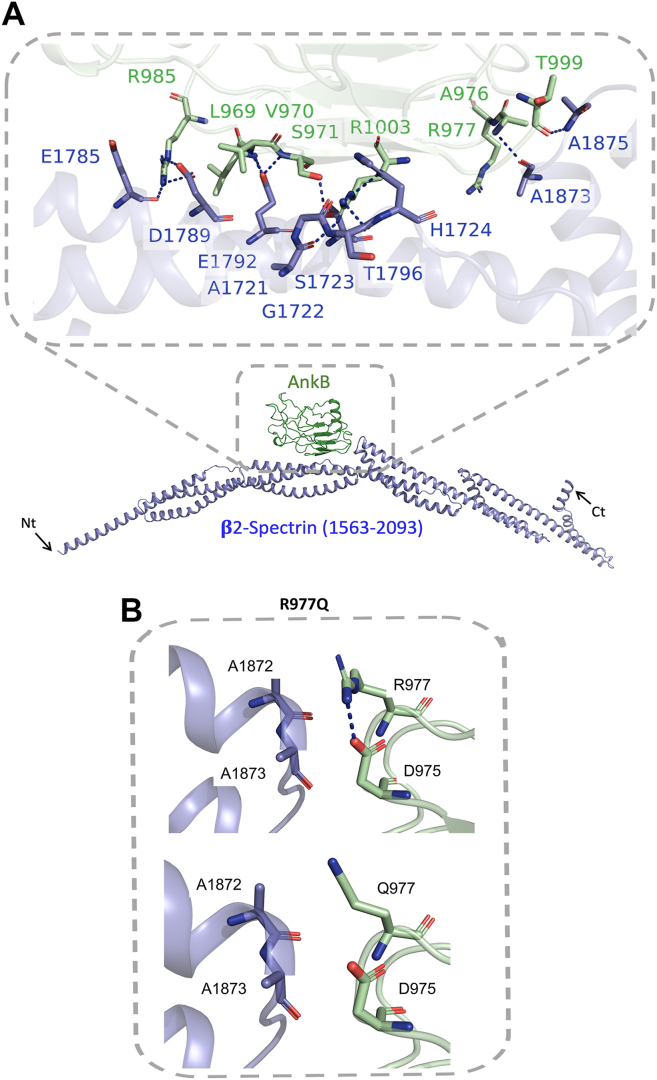
**AlphaFold Model of the AnkB Spectrin-Binding Domain Interaction with β2-Spectrin.***A*. AlphaFold-predicted structure of the AnkB/β2-Spectrin complex, showing the AnkB spectrin-binding domain (residues 966–1125, *green*) bound to spectrin repeats 14 and 15 of β2-Spectrin (residues 1563–2093, *blue*). *Arrows* indicate the N- and C-terminal ends (Nt, Ct). *Dashed blue lines* represent H-bonds. *B*. Structural model generated using the PyMOL mutagenesis tool illustrating the intramolecular salt bridge between AnkB (*green*) residues R977 and D975 in the SBD, and β2-Spectrin (*blue*) at residues A1872 and A1873 in spectrin repeat R15. The ASD-associated AnkB 977Q mutation disrupts the intramolecular interaction, weakening its binding to β2-Spectrin. The *dashed blue line* represents a salt bridge. AnkB, ankyrin B; SBD, spectrin-binding domain.

Spectrin β1-4 proteins are present in spines and dendrites of pyramidal neurons, and β2-Spectrin (UniProt ID: Q01082) is the most robustly expressed isoform at these sites (62, 63, 64, 65). To elucidate the molecular interactions stabilizing the AnkB/β2-Spectrin complex and assess the impact of ASD-associated variants in ANK2, we employed AlphaFold2 (version 2.2; https://github.com/google-deepmind/alphafold) to model the complex, focusing on the SBD of AnkB (residues 966–1125) and β2-Spectrin’s repeats 14 and 15 (residues 1563–2093) (66). Five AlphaFold2 models were generated, revealing a consistent interaction interface with minor variations in loop conformations (Fig. 5*A*, Table S3). Key interactions included H-bonds between AnkB residues (*e.g.*, Ser971, Arg985, Arg1003, Thr999) and β2-Spectrin residues (*e.g.*, Ala1721, Ser1723, Glu1785, Asp1789, Thr1796, Ala1873, Ala1875), alongside a stable salt bridge between Arg985 (AnkB) and Asp1789 (β2-Spectrin) (Table S3). The models exhibited high reliability, with pTM and ipTM scores of 0.71 and 0.69, respectively, and RMSD values ranging from 0.364 to 1.146 Å across the five models, indicating structural consistency.

**Supporting Figure S7:**
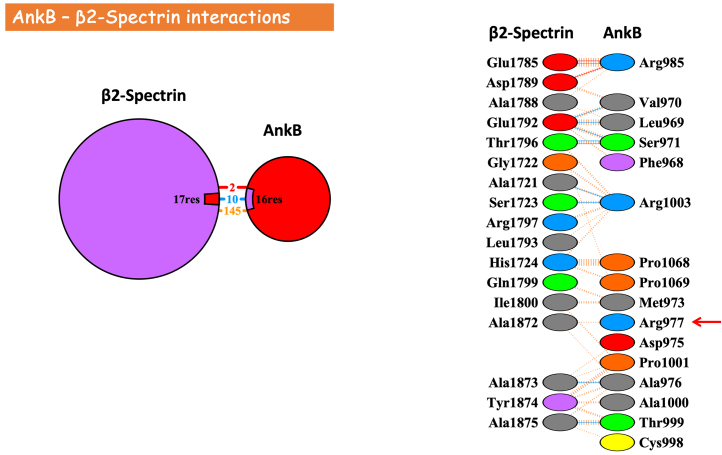


Residue-level interaction analysis using PDBsum revealed a robust interface comprising two salt bridges, 10 H-bonds, and 145 non-bonded contacts between AnkB and β2-Spectrin (Fig. S7). Table S4 details these interactions, including interaction types and distances, highlighting their role in stabilizing the complex. Structural comparison with the AnkB structure (PDB ID: 4RLV) showed that the β2-Spectrin-binding interface is spatially distinct from the auto-inhibitory segment interacting with AnkB repeats R1–R14. This separation suggests that β2-Spectrin binding does not compete with the auto-inhibitory segment, allowing independent regulation of AnkB’s interactions. These findings provide a structural basis for understanding how ASD-associated variants in ANK2 may disrupt the AnkB/β2-Spectrin complex, consistent with experimental mutagenesis data.

**Figure 6:**
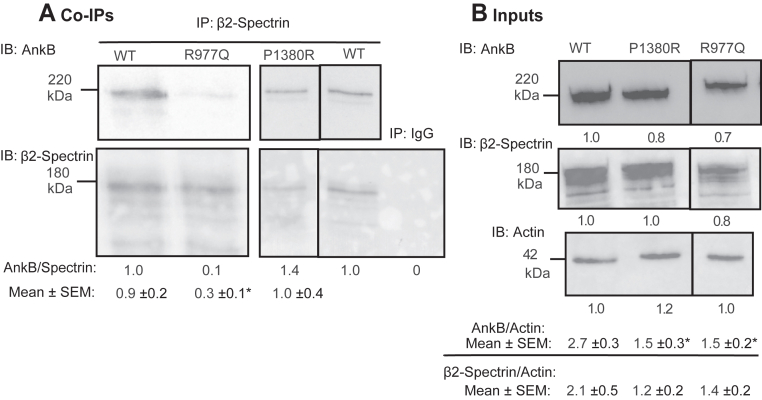
**Altered Interaction of ASD Missense Mutation in AnkB R977Q with β2-Spectrin.***A*. AnkB-220 co-immunoprecipitated with GFP-tagged β2-Spectrin from transfected HEK293 cells using anti-GFP antibodies. Inherited ASD mutation AnkB R977Q showed significantly decreased association with β2-Spectrin, but *de novo* mutation AnkB P1380R was not impaired for binding. AnkB/β2-Spectrin ratios in the immunoprecipitates relative to WT are shown under the representative blots. Lanes from the same blot are shown without a line, or with a line if not adjacent. Blots from different gels are shown with a wider separation. Mean ratios of AnkB/β2-Spectrin ± SEM from n replicate experiments are indicated below. Mean differences (2-tailed *t* test, ∗*p* < 0.05) were significant between WT (n = 12) and ∗ R977Q (n = 7, *p* = 0.02) but not for P1380R (n = 6, *p* = 0.82). *B*. Input WT and mutant AnkB-220 levels in HEK293 lysates (5 μg) prior to immunoprecipitation were determined by IB with anti-HA antibodies, followed by stripping and reprobing with anti-EGFP antibodies, and anti-Actin antibodies as loading controls. Lanes from the same blot are shown without a line or with a line if not adjacent. Levels of AnkB, β2-Spectrin, and Actin relative to WT in the representative blots are shown below each lane. Mean ratios of AnkB/Actin ± SEM and β2-Spectrin/Actin from n replicate experiments are indicated below. There was a significant mean difference in AnkB/Actin (2-tailed *t* test, ∗*p* < 0.05) between WT (n = 4) and mutants R977Q (n = 4, *p* = 0.02) and P1380R (n = 4, *p* = 0.02).There was not a significant mean difference in β2-Spectrin/Actin between WT and R977Q (*p* = 0.31) or P1380R (*p* = 0.17). The position of the 220 kDa molecular weight marker from PageRuler Plus is indicated and coincides with the AnkB220-HA band. AnkB, ankyrin B; IB, immunoblotting.

**Supporting Figure S8:**
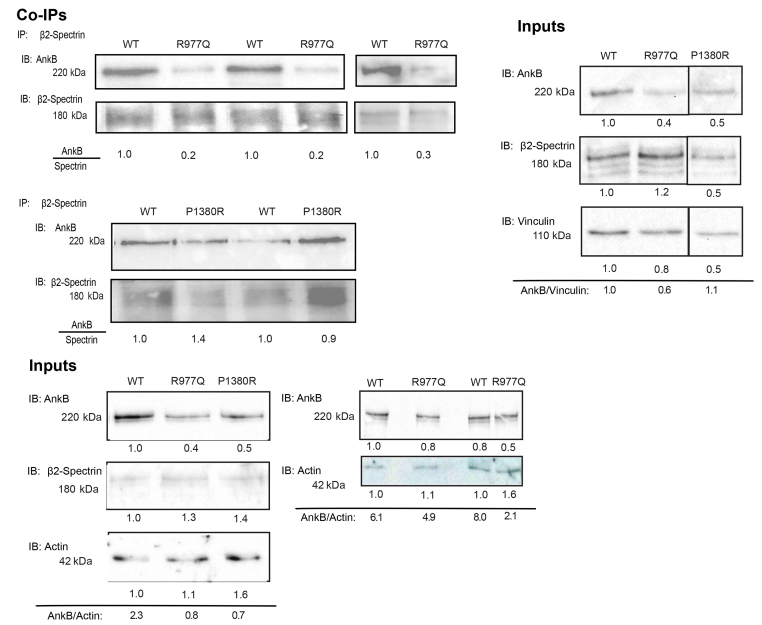


We next assessed protein-protein interactions of two ASD missense mutations predicted to impact β2-Spectrin binding in the AnkB SBD: a maternally inherited mutation R977Q in the ZU5A region (4) and *de novo* mutation P1380R in the UPA region (47). We generated the two mutations in HA-tagged AnkB-220 cDNA and co-expressed them with β2-Spectrin-EGFP cDNA in HEK293 cells. β2-Spectrin-EGFP was pulled down from cell lysates with anti-EGFP antibodies adsorbed to Protein A/G magnetic beads. Immune complexes were analyzed by Western blotting with anti-HA antibodies, followed by re-probing with anti-GFP antibodies. Relative levels of β2-Spectrin/AnkB were quantified by densitometry. AnkB-220 R977Q was impaired for binding to β2-Spectrin, as evident from the decreased AnkB/β2-Spectrin-GFP ratio in co-immunoprecipitates relative to WT (Fig. 6*A*). Quantification of replicate blots (Fig. S8) yielded mean NrCAM/AnkB ratios (±SEM) that were significantly decreased for AnkB R977Q compared to WT (Fig. 6*A*; two-tailed *t* test, ∗*p* < 0.05). Structural analysis using AlphaFold2 models provided insights into the impact of the ANK2 R977Q mutation on AnkB/β2-Spectrin binding, rationalizing the reduced complexation observed in immunoprecipitation experiments (Fig. 5*B*). The model suggests that the R977Q mutation disrupts a salt bridge between AnkB Arg977 and β2-Spectrin Asp975, while potentially forming a H-bond with Asp975 that is less stabilizing due to altered charge interactions. Additionally, the mutation weakens hydrophobic interactions with β2-Spectrin Ala1872 and Ala1873. These structural changes are consistent with experimental mutagenesis data showing reduced AnkB/β2-Spectrin binding. In contrast, AnkB-220 P1380R did not exhibit decreased affinity for β2-Spectrin in the co-immunoprecipitation assay, as evident from mean AnkB/β2-Spectrin ratios in immunoprecipitates in replicate assays (Figs. 6*A* and S8). The P1380R mutation is located C-terminally in a disordered region outside the AlphaFold modeled region of the AnkB SBD.

To evaluate whether these AnkB mutant proteins were stably expressed, lysates of HEK293 cells were transfected with WT or mutant AnkB plasmids, together with equimolar levels of β2-Spectrin-EGFP cDNA, and equal amounts of cell lysate protein were analyzed by Western blotting. The two ASD-related AnkB mutants were expressed at reduced levels compared to WT AnkB relative to Actin as shown in the representative blot (Fig. 6*B*). Replicate experiments yielded similar results (Fig. S8) with mean AnkB/Actin or AnkB/Vinculin ratios (±SEM) that were approximately 60% of WT (Fig. 6*B*; two-tailed *t* test, ∗*p* < 0.05). β2-Spectrin levels were similar in mutant versus WT expressing cells relative to Actin (Figs. 6*B* and S8). These results suggested that the AnkB R977Q and P1380R proteins are partly unstable in HEK293 cells.

### Sema3F-induced spine retraction is promoted by WT AnkB-220 but not by AnkB-A368G

**Figure 7:**
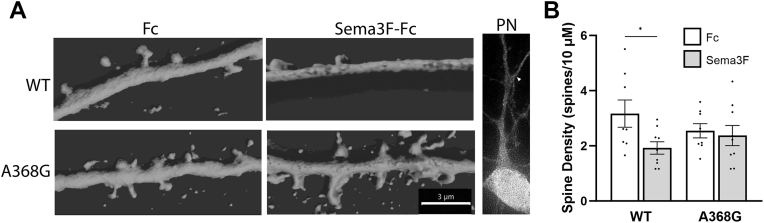
**Sema3F-Induced Spine Retraction is Impaired in Neuronal Cultures from *Ank2*^*+/−*^ Mice Expressing the ASD Mutation AnkB A368G.***A, Ank2*^*+/−*^ cortical neuronal cultures were transfected with pCAG-IRES-EGFP together with plasmids expressing WT AnkB-220 or AnkB-220 mutant A368G. Cells were treated on DIV14 with 5 nm Fc (*control*) or Sema3F-Fc for 30 min, and immunostained for EGFP. Representative confocal images of EGFP-labeled apical dendrites on pyramidal neurons were subjected to 3D reconstruction in Imaris. This scale bar = 3 μm, all panels. Lower magnification confocal image of pyramidal neuron with arrowhead pointing to the first branch of the apical dendrite. *B*, quantification of mean spine density ± SEM per neuron on apical dendrites in neuronal cultures described in A. Each point presents the mean spine density per neuron per 10 μm length of apical dendrite. Results show that Sema3F-Fc induces significant spine pruning relative to Fc controls in *Ank2*^*+/−*^ cortical neurons expressing WT AnkB but not ASD mutant AnkB A368G. *p*-values for Sema3F-Fc versus Fc (two tailed *t* test, unequal variance) were for WT (∗*p* = 0.04) and A368G (*p* = 0.71). n = 123 to 172 spines on 10 pyramidal neurons per condition. AnkB, ankyrin B; Sema3, 3 Semaphorins.

Studies in transgenic mouse models and neuronal cultures have demonstrated that Sema3s trigger spine retraction on apical dendrites through NrCAM, Neuropilin-2, and AnkB-220 (21, 22, 23, 30, 67). Because AnkB A368G was the most compromised ASD missense mutation for NrCAM interaction, we assessed whether it was deficient in spine pruning using an established assay for Sema3F-induced dendritic spine retraction in cultured cortical neurons from Ank2 mutant mice. Neurons from *Ank2*^*+/−*^ heterozygotes and *Ank2*^*−/−*^ homozygotes have been shown to be inhibited for Sema3F-induced spine retraction in this *in vitro* assay (30). Cortical neuronal cultures from *Ank2*^*+/−*^ heterozygous embryos (E15.5) were co-transfected on DIV11 with AnkB-220 WT or mutant cDNA together with pCAG-IRES-mEGFP plasmids. Neurons in replicate cultures were treated on DIV14 with Sema3F-Fc or control Fc (5 nM) for 30 min and spine density quantified on apical dendrites in deconvolved confocal z-stacks using Neurolucida software (https://mbfbioscience.com/products/neurolucida/). Mean spine density of neurons expressing WT AnkB-220 or mutant A368G AnkB-220 were compared by the *t* test (two-tailed, *p* < 0.05). Neurons transfected with WT AnkB-220 responded to Sema3F-Fc with spine retraction that was significantly greater than that of control Fc treated neurons (Fig. 7). In contrast, neurons expressing A368G AnkB-220 did not respond to Sema3F-Fc with significant spine retraction (Fig. 7). Representative images of apical dendrites from WT or mutant transfected cultures treated with Fc or Sema3F-Fc are shown in 3D reconstructions performed in Imaris (Fig. 7), and as raw confocal images (Fig. S9).

### Pathogenicity of AnkB variants associated with ASD

As shown in Table S5, the AlphaMissense database revealed pathogenicity scores (threshold ≥0.7 for pathogenic) that were significant for AnkB variants A368G, A373V, I807M, R977Q, and P1380R. Variants A525V and E819K were benign or ambiguous. REVEL scores (threshold ≥0.65 for pathogenic) for available variants were also significant for A368G (0.682, pathogenic), A373V (0.723, pathogenic), I807M (0.876, pathogenic), R977Q (0.891, pathogenic), and P1380R (0.943, pathogenic) but not for A525V (0.421, benign) or E819K (0.598, ambiguous). Allele frequencies from gnomAD v3.1.2 were negligible for maternally inherited variants (*e.g.*, A368G: 1.3 × 10^−5^, A373V: not observed) and absent for *de novo* variants (I807M, P1380R), consistent with their rarity. According to the Human Gene Mutation Database (HGMD) (68). AnkB variants A368G, A525V, I807M, E819K, and P1380R are associated exclusively with ASD and not with intellectual disability, bipolar disorder, or seizures. Variants A373V and R977Q were reported linked to ASD (47). This data confirms the rarity and likely pathogenicity of the variants which displayed altered binding characteristics, and support their relevance to ASD.

## Discussion

AnkB regulates dendritic spine and excitatory synapse density of cortical pyramidal neurons, and mediates Sema3F-induced spine pruning through NrCAM (30). Molecular modeling of the AnkB MBD in complex with the NrCAM cytoplasmic domain using AlphaFold defined a binding pocket in the R11 ANK repeat of AnkB for the NrCAM cytoplasmic sequence QFN^1267^E^1268^DGSFIGQY^1276^, which is highly conserved in the L1-CAM family. It is worth mentioning here that the auto-regulating disordered segment of AnkB plays a critical role in modulating its interactions with binding partners. In the auto-inhibited state (PDB ID: 4RLV), this segment binds to the AnkB repeat core, sterically hindering access to binding sites for NrCAM. We hypothesize that displacement of this segment, potentially through post-translational modifications or competitive binding, activates AnkB. Critical residues R297, R308, and H374 in the AnkB MBD were identified that engaged NrCAM residues E1268, N1267, and Y1276, respectively, at the binding interface. Substitution mutagenesis of these AnkB residues resulted in significant losses of NrCAM binding in co-immunoprecipitation assays. AlphaFold modeling predicted that the inherited ASD mutation AnkB A368G is within a α-helical segment of the MBD near the NrCAM binding interface. The AnkB A368G mutation markedly disrupted association with NrCAM, despite being stably expressed. In contrast the ASD missense mutation AnkB A373V, located within the NrCAM binding interface adjacent to H374, significantly increased binding to NrCAM. Increased stability could influence molecular dynamics of the AnkB/NrCAM complex or phosphorylation of Y1276 in the FIGQY motif, which promotes Ankyrin dissociation from L1-CAMs (34). Other AnkB ASD missense mutations at or near the MBD-A525V, I807M, and E819K-were stably expressed and had little effect on NrCAM binding. These variants were not predicted in our models to exhibit a binding interface with NrCAM. ASD pathology associated with these *Ank2* mutations could be due to altered interactions with other binding partners, or to other genes.

The AnkB A368G mutation was functionally impaired for Sema3F-induced spine retraction in mouse cortical neuron cultures. Previous structure-function studies demonstrated that binding between the extracellular domains of NrCAM and Npn-2 increases the affinity of Npn-2 for PlexA3, thus promoting signaling through Rap-GAP and spine pruning (24). AnkB binding to the NrCAM cytoplasmic domain may enhance stability of the Sema3F holoreceptor NrCAM/Npn-2/PlexA3 at the neuronal membrane by linking it to the spectrin-actin cytoskeleton. Although consequences of AnkB A368G *in vivo* have not been determined, loss of NrCAM/AnkB binding could contribute to the ASD phenotype by inhibiting developmental spine pruning, consistent with increased spine density in frontal brain regions in ASD (14, 15, 16, 17, 18).

AlphaFold modeling of the AnkB/β2-Spectrin complex identified key interactions between the AnkB SBD and β2-Spectrin repeats 14 and 15. β2-Spectrin variants have been linked to ASD and related syndromes (45, 53, 54). The ASD missense mutation (R977Q) in the Zu5A subdomain of the AnkB SBD disrupted association with β2-Spectrin. In contrast, the ASD missense mutation P1380R was not impaired for β2-Spectrin binding. Both AnkB R977Q and P1380 exhibited reduced levels of expression in HEK293 cells. This decrease in stability might impact known β2-Spectrin functions in dendrite development, cell membrane organization, or transport (reviewed in (45)). However, the stability of these interactions in neurons or consequences on neuronal function has not been evaluated.

AnkB ASD mutations might alter other critical functions of L1-CAMs than spine pruning. For example, AnkB and NrCAM cooperate in stabilizing synaptic contacts made by cholecystokinin basket interneurons onto pyramidal cell soma (69). Ankyrins also stabilize GABAergic inputs onto pyramidal cell soma through L1 (39). AnkB interaction with Neurofascin promotes axonal-glial interactions at paranodes of myelinating Schwann cells (70). AnkB also localizes ion channels and exchange proteins at the cardiomyocyte membrane (71). The function of other Ankyrins might also be affected by homologous mutations. For example, L1 binding to AnkG drives chandelier interneuron innervation of the axon initial segment of pyramidal cells in cortex (40) and cerebellum (72). Additionally, AnkR organizes L1-CAMs, perineural nets, and K+ channels through β1-Spectrin in parvalbumin interneurons (73). A consideration is that all of the mutations in the present study target both AnkB-220 and AnkB-440, and thus increased axonal branching and ectopic connectivity due to AnkB-440 deficiency could also be impacted (31, 32).

In summary, these new findings provide insight into the L1-CAM/AnkB/β2-Spectrin complex and molecular basis of ASD etiology associated with AnkB missense mutations.

## Experimental procedures

### Structural modeling of AnkB-NrCAM and AnkB-β2-spectrin complexes

The structural models of AnkB and its interactions with NrCAM and β2-Spectrin were predicted using AlphaFold v2.2 (74). We modeled specific domains of AnkB, NrCAM, and β2-Spectrin to focus on regions known to be implicated in their interactions. Modeling full-length proteins was computationally infeasible due to their large sizes (>2000 residues each) and would likely introduce noise from disordered or non-interacting regions. The cytoplasmic domain of human NrCAM (residues 1191–1304, UniProt ID: Q92823) was modeled in association with ANK repeats 1 to 24 in the human AnkB MBD (residues 30–822, UniProt ID: Q01484). Similarly, the complex between AnkB human β2-Spectrin (UniProt ID: Q01082) was modeled, focusing on the interface between AnkB residues 966 to 1125 and β2-Spectrin residues 1563 to 2093, based on the crystal structure of human β2-Spectrin. We generated five models for each complex using AlphaFold2. To enhance the accuracy of the predicted models, the complex structures were further optimized using the AMBER molecular dynamics package, employing energy minimization and equilibration protocols (75). We performed energy minimization using AMBER MD (5000 steps, steepest descent) with position restraints (10 kcal/mol/Å^2^) on backbone atoms to maintain the predicted conformation.

Interactions between AnkB and NrCAM or β2-Spectrin were visualized using The PyMOL Molecular Graphics System, Version 3.0 (Schroedinger, LLC.) and Chimera-X (76). The PDBsum tool (77) was utilized to identify detailed inter-protein interactions. Electrostatic interactions and the charged surface distributions of the complexes were analyzed using Advanced Poisson-Bolzmann Solver software (APBS; https://www.poissonboltzmann.org/) (78). The PyMOL mutagenesis tool was employed to model specific mutations in AnkB, NrCAM, and β2-Spectrin. These mutations were structurally analyzed to evaluate their impact on binding interfaces and overall complex stability.

### Plasmids and mutagenesis

Plasmids used were: *Ank2* cDNA encoding the human AnkB isoform of 220 kDal molecular weight with a C-terminal 2× hemagglutinin (HA) tag in pEGFP-N1-ΔEGFP (Addgene), mouse NrCAM cDNA (NCBI nm_01127493; variant three in pCMV6-XL4 (21)), human β2-Spectrin with C-terminal EGFP tag in pEGFP-N1 (79). Mutations were engineered into AnkB-220 by site-directed mutagenesis using Q5 mutagenesis (New England Biolabs). Sequences were confirmed by whole plasmid sequencing at Plasmidsaurus, Inc.

### Immunoreagents

Antibodies used were directed against the following proteins: rabbit anti-NrCAM (Abcam #24344 or R&D Systems AF8538), chicken anti-EGFP (Abcam #13970), rabbit anti-GFP (Invitrogen A6455), mouse anti-AnkB (Invitrogen 33-3700), mouse anti-Actin (EMD Millipore Ab1501), mouse anti-HA (Life Technologies, 2-2.2.14). Non-immune rabbit IgG (NIgG), HRP- and AlexaFluor 488, 555, and 594-conjugated secondary antibodies were from Jackson Immunoresearch.

### HEK293 cell culture, transfection, and immunoprecipitation

HEK293 cells were grown in DMEM/gentamicin/kanamycin/10% FBS in a humidified incubator with 5% CO_2_. Cells were seeded at 2 × 10^6^ cells/100mm dish and transfected when cells reached ∼ 2 × 10^7^ cells/dish. AnkB 220-2x HA and NrCAM plasmids were transfected in equimolar quantities using Lipofectamine 2000 in Opti-MEM as described (22). Media was changed to DMEM after 18 h, and cells were lysed and collected 48 h post-transfection. Cells were harvested in Brij98 lysis buffer (1% Brij98, 10 mM Tris-Cl pH 7.0, 150 mM NaCl, 1mM EDTA, 1mM EGTA, 10 mM NaF, protease inhibitors (SigmaAldrich).

For co-IP of NrCAM and AnkB-220-HA, lysates of transfected HEK293 cells (0.5 mg) were diluted in co-IP binding buffer (PBS, 0.1% TritonX-100, protease inhibitors) and precleared by incubating with Pierce Protein A/G magnetic beads (Thermo Fisher Scientific) for 30 min at 4 °C. Precleared lysates (duplicates or triplicates, 500 ug each) were then incubated with fresh Protein A/G magnetic beads that had been coated with mouse anti-HA antibody or control NIgG overnight at 4 °C. Beads were collected using a magnetic stand and washed 4× in co-IP binding buffer. Immune complexes were eluted in SDS sample buffer by boiling for 2 min. Proteins were separated by SDS-PAGE (6%) and transferred to nitrocellulose membranes. Membranes were blocked in TBST (Tris buffered saline/0.1% Tween-20) containing 5% nonfat dried milk and incubated overnight with primary antibodies (1:1000), washed, and incubated with HRP-secondary antibodies (1:5000) for 1 h. Blots were first probed with rabbit polyclonal antibody to NrCAM (R&D 8538) and HRP-conjugated donkey anti-rabbit IgG. All antibodies were diluted in 5% nonfat dry milk/TBST. Blots were developed using Western Bright ECL Substrate (Advansta). Images were captured with a BioRad ChemiDoc imager for times yielding a linear response of signal. Blots were then stripped and reprobed for AnkB using mouse anti-HA monoclonal antibody and HRP-conjugated goat anti-mouse IgG. Densitometry of bands on TIFF images was measured in FIJI (mean gray value, 8 bit). Background was subtracted using the rolling ball method with radius set to 50 pixels. The ratio of AnkB to NrCAM was calculated from replicate experiments, and means ± SEM compared between WT and mutants by the *t* test (two-tailed, unequal variance, *p* < 0.05). WT and mutant bands on the same representative blot were normalized to WT.

For co-immunoprecipitation of AnkB-220-HA and β2-Spectrin-GFP, lysates of HEK293 cells (0.5 mg) transfected with equimolar quantities of plasmids were diluted in co-IP binding buffer and precleared by incubating with Protein A/G magnetic beads for 30 min at 4 °C. Beads were removed using the magnetic stand. Precleared lysates (duplicates or triplicates, 500 ug each) were incubated with rabbit-anti-EGFP antibody or control IgG for 4 h at 4 °C, then with Protein A/G magnetic beads. Beads were collected using the magnetic stand and washed 4× in co-IP binding buffer. Immune complexes were eluted in SDS sample buffer, separated on a 6% SDS-PAGE gel and transferred to nitrocellulose membranes. Membranes were immunoblotted with mouse anti-HA antibody and HRP-conjugated goat anti-mouse IgG, and imaged on the ChemiDoc. Membranes were stripped and reprobed for β2-Spectrin-GFP using rabbit anti-GFP and HRP-donkey anti-rabbit IgG. Densitometry of bands on TIFF images was measured and background subtracted, as described above. The ratio of AnkB to β2-Spectrin-GFP was determined from replicate experiments, and means ± SEM compared between WT and mutants by the *t* test (*p* < 0.05). Bands on representative blots were normalized to WT in figures.

For assaying the relative levels of WT and mutant AnkB in input HEK293 cell lysates prior to co-immunoprecipitation, equal amounts of protein (5 μg) determined by BCA protein analysis were subject to SDS-PAGE, transferred to nitrocellulose, and immunoblotted with the indicated antibodies. Membranes were reprobed first with antibodies to NrCAM or β2-Spectrin-GFP, followed by anti-Actin antibodies as loading controls. Images were collected with the BioRad ChemiDoc imager for times producing a linear response of signal. Densitometric signals of bands on representative blots were normalized to WT in the figures. Ratios of WT and AnkB relative to Actin were calculated and means ± SEM were compared between WT and mutants by the *t* test (2-tailed, *p* < 0.05).

### Mouse cortical neuron cultures and Sema3F-induced spine retraction

We previously described the production of cortical neuron cultures from *Ank2*-null and heterozyous mice on a first generation Sv129/C57Bl hybrid background (30). Cortical neurons from homozygous *Ank2*^−/−^ and heterozygous *Ank2*^+/−^ hybrids exhibited decreased spine collapse in response to Sema3F-Fc, which could be rescued by transfection of cDNA encoding WT AnkB-220 (30). *Ank2* heterozygous mice on C57BL/6 (80) were crossed with WT Sv129 mice to produce *Ank2*-heterozygous F1 hybrids. Intercrossing these F1 hybrids yielded WT, *Ank2*^+/−^, and *Ank2*^−/−^ genotypes. Because *Ank2*^−/−^ embryos were present in lower than Mendelian ratios, we focused on *Ank2*-heterozygous neurons. Mice were maintained according to policies of the University of North Carolina Institutional Animal Care and Use Committee and approved by that committee (IACUC; AAALAC Institutional Number: #329; ID# 18-073, 21-039) in accordance with NIH guidelines.

Cortical neurons were plated onto Lab-Tek II chamber slides (1.5 × 10^6^ cells/well) coated with poly-D-lysine and laminin as described (22). Genotyping of embryos was performed on tail DNA after plating the cells. AraC was added at 5 days *in vitro* (DIV5) to limit the growth of glia and fibroblasts, and media was changed on DIV7. At DIV11, cells were transfected with pCAG-IRES-mEGFP with WT or mutant AnkB-220 plasmids using Lipofectamine 2000 (22, 24). At DIV 14, replicate cultures were treated with purified Fc from human IgG (Abcam #ab90285) or recombinant Sema3F-Fc fusion protein (R&D Systems #3237-S3) at 5 nM for 30 min. Cultures were fixed with 4% paraformaldehyde (PFA), quenched with 0.1M glycine, permeabilized with 0.1% Triton X-100, and blocked with 10% donkey serum. Cells were incubated with chicken anti-GFP primary antibody and AlexaFluor AF488-conjugated goat anti-chicken secondary antibody (1:500), washed, and mounted. At least 10 images of apical dendrites of labeled pyramidal neurons were captured per condition using a Zeiss Axioplan epifluorescence microscope equipped with a 40X oil objective. Images were deconvolved for scoring spine density on apical dendrites. Apical dendrites were differentiated from basal dendrites by cell morphology. Pyramidal neurons extend a single prominent, thicker apical dendrite from the apex of the pyramidal-shaped soma, whereas they extend multiple thinner basal dendrites from the base of the soma (27). Any ambiguous dendrites were not analyzed. Spines were scored blind to observer using Neurolucida software as described (30). Mean spine densities (number/10 μm ± SEM) were calculated and compared for significant differences by the *t* test (2-tailed, unequal variance, *p* < 0.05). In some instances confocal z-stacks were captured on a Zeiss LSM900 microscope using a Plan-apochromat 63 × 1.4 numerical aperture objective and Zen software (https://www.zeiss.com/microscopy/en/products/software/zeiss-zen.html) in 0.2 μm optical sections. 3D reconstructions were rendered from dendritic z-stacks with Imaris (Bitplane; https://imaris.oxinst.com/) software. All experiments were designed to provide sufficient power (80–90%) to discriminate significant differences (*p* < 0.05) in means (±SEM) between independent controls and experimental samples as described (81). The type I error probability associated with tests of the null hypothesis was set at 0.05.

## Data availability

All predicted AlphaFold models have been deposited in Zenodo and are available under https://doi.org/10.5281/zenodo.17065053. Other datasets supporting the conclusions of this study are included in the article and its supporting information files. Additional information is available from the corresponding author upon reasonable request.

## Supporting information

This article contains supporting information.

## Conflict of interest

The authors declare they have no conflicts of interest with the contexts of this article.

## Figures and Tables

**Supporting Figure S9:**