# Supplementary material for: Structural interactions of ankyrin B with NrCAM and β2 spectrin
Source: J Biol Chem. 2025 Oct 30;301(12):110872. doi: 10.1016/j.jbc.2025.110872 (PMC12681835; doi:10.1016/j.jbc.2025.110872)
Supplement: Supporting Figure legends [file mmc1.docx]

Supporting Figure Legends

Figure S1: Interaction Analysis of AnkB and NrCAM by PDBSum

PDBSum analysis of ANK repeats 1-24 of the AnkB MBD with the NrCAM cytoplasmic domain identified 8 salt bridges (red lines), 18 hydrogen bonds (blue lines), and 415 non-bonded contacts (orange dashed lines) between the indicated residues. Residue colors: positively charged (H,K,R-blue), negatively charged (D,E-red), neutral (S,T,N,Q-green), aliphatic (A,V,L,I,M-gray), aromatic (F,Y,W-purple), (P,G-orange). Arrows indicate the AnkB residues that were mutagenized and tested for NrCAM binding.

Figure S2: Confidence Analysis of the AlphaFold-Predicted AnkB/NrCAM Complex

Structural model of the AnkB/NrCAM complex colored by per-residue pLDDT (predicted Local Distance Difference Test) score, with AnkB membrane binding domain (residues 30–822) and NrCAM cytoplasmic domain (residues 1191–1304) shown. The color gradient ranges from blue (pLDDT > 90, high confidence) to red (pLDDT < 50, lower confidence), highlighting regions of variable structural reliability. At right, Graph of Expected Position Error (Å) for the AnkB/NrCAM complex (AnkB residue numbers in black; NrCAM residue numbers in magenta) plotted as a function of residue position. The plot indicates predicted positional accuracy across the model, with lower expected position error values corresponding to higher confidence regions.

Figure S3: AnkB Associates with NrCAM in a Nexus of Residues in ANK Repeat R11

**Co-IPs**: Additional Western blots showing that NrCAM co-immunoprecipitates with WT AnkB-220 (HA-tagged) from transfected HEK293 cells, and to a much lesser extent with HA- AnkB-220 with point mutations H374W, H374A, R308E, and R297E.

AnkB was immunoprecipitated (IP) from cell lysates with anti-HA antibodies and immunoblotted (IB) with anti-NrCAM antibodies, followed by reprobing with anti-HA antibodies. NrCAM/AnkB ratios in the immunoprecipitates were obtained by densitometry relative to WT. Lanes from the same blots are shown without a line. Blots from different gels are shown with a wider separation. Nonimmune IgG did not pull down AnkB or NrCAM.

**Inputs:**  Additional Western blots of WT and mutant HA-AnkB-220 in HEK293 lysates (5 µg) prior to immunoprecipitation as determined by immunoblotting (IB) with anti-HA antibodies, followed by stripping and reprobing with anti-NrCAM antibodies. Blots from different gels are separated. Levels of mutants relative to WT are shown below each lane. AnkB/Actin ratios of mutants relative to WT in inputs are shown (actin blots of lysates are shown in Fig. 3).

The position of the 220 kDa molecular weight marker from PageRuler Plus is indicated

and coincides with the AnkB220-HA band.

Figure S4: Altered Interactions of ASD Missense Mutations AnkB A368G and A373V with NrCAM

**Co-IPs**: Additional Western blots showing that NrCAM co-immunoprecipitates with WT HA-AnkB-220 from HEK293 cells, and to lesser extent with HA-AnkB-220 with the mutation A368G. HA-AnkB-220 with mutation A373V shows increased binding to NrCAM. AnkB was immunoprecipitated (IP) from cell lysates with anti-HA antibodies, and immunoblotted (IB) with anti-NrCAM antibodies followed by reprobing with anti-HA antibodies. NrCAM/AnkB ratios in the immunoprecipitates were obtained by densitometry relative to WT on each gel. Lanes on the same blots are shown with or without a line. Blots from different gels are shown with a wider separation. Nonimmune IgG did not pull down AnkB or NrCAM.

**Inputs:**  Additional Western blots of WT and mutant HA-tagged AnkB in HEK293 lysates (5 µg) prior to immunoprecipitation as determined by immunoblotting (IB) with anti-HA antibodies, followed by stripping and reprobing with anti-NrCAM antibodies, and anti-Actin antibodies. Blots from different gels are separated. Levels of mutants relative to WT on each gel are shown under the lanes. The AnkB/Actin ratio of mutants relative to WT on each gel is shown below.

Figure S5: Structural analysis of AnkB A373V mutation in the AnkB/NrCAM complex

(A) Structural view of the AlphaFold-predicted AnkB/NrCAM complex showing wild-type AnkB A373, where interactions with NrCAM Y1276 (FIGQY motif) via AnkB I404 are minimal.

(B) Structural view of the AnkB A373V mutant, highlighting V373 (purple) forming enhanced hydrophobic interactions (brown dots) allosterically contacting NrCAM Y1276 (magenta) via AnkB I404, stabilizing the complex.

Figure S6: Comparison of residue interaction patterns and structural alignments in AnkB/NrCAM and experimentally resolved Ankyrin complexes.

1. Residue-level interaction diagrams comparing the AlphaFold2-predicted AnkB/NrCAM complex with experimentally resolved Ankyrin complexes (PDB IDs: 4RLY, 5Y4F, and 5Y4D). Interactions are visualized using the PDBsum color scheme: blue for H-bonds, red for salt bridges, and orange for hydrophobic interactions. Amino acids are colored by physicochemical properties: positively charged residues (blue), negatively charged residues (red), neutral residues (green), aliphatic residues (gray), aromatic residues (pink), Proline and Glycine (orange), and Cysteine (yellow). (B) Structural superposition of the AnkB domains from the AlphaFold2-predicted AnkB/NrCAM complex and experimentally resolved structures (PDB IDs: 4RLY, 5Y4F, and 5Y4D). Each complex is distinctly colored: AnkB/NrCAM (magenta), 4RLY (AnkG/Nav1.2, green), 5Y4F (AnkB/AI-c, red), and 5Y4D (AnkG/AnkR chimera, blue). (C) Sequence alignment of the NrCAM cytoplasmic domain (residues 1191–1304) and the AnkB auto-inhibitory N-terminal segment (PDB ID: 4RLV). Identical residues are highlighted in red blocks, with secondary structural elements indicated above and below the alignment for clarity.

Figure S7: Interaction Analysis of AnkB and β2-Spectrin by PDBSum

PDBSum analysis of AnkB residues 966-1125 in the SBD with β2-Spectrin residues 1563-2093 in spectrin repeats 14 and 15 identified 2 salt bridges (red lines), 10 hydrogen bonds (blue lines), and 145 non-bonded contacts (orange lines) between the indicated residues. Residue colors: positively charged (H,K,R-blue), negatively charged (D,E-red), neutral (S,T,N,Q-green), aliphatic (A,V,L,I,M- gray), aromatic (F,Y,W-purple), (P,G-orange), (C-yellow). Arrow indicates AnkB R977, which is mutated in ASD variant AnkB R977Q.

Figure S8: Altered Interaction of ASD Missense Mutation AnkB R977Q with β2-Spectrin

**Co-IPs:** Additional Western blots of AnkB-220 co-immunoprecipitated with GFP-tagged β2-Spectrin from HEK293 cell lysates using anti-GFP antibodies. ASD mutation AnkB R977Q showed decreased association with β2-Spectrin, but AnkB P1380R was not affected. NrCAM/β2-Spectrin ratios in immunoprecipitates relative to WT on the same gels were obtained by densitometry. Lanes on the same blots are shown without separation lines. Blots from different gels are shown with a wider separation.

**Inputs**: Additional Western blots of WT and mutant AnkB-220 in HEK293 lysates (5 µg) were determined by immunoblotting (IB) with anti-HA antibodies, followed by stripping and reprobing with anti-EGFP antibodies, anti-Actin antibodies or anti-Vinculin antibodies as loading controls. Levels of mutants relative to WT are shown below each lane.

Lanes from the same blot are shown without a line, or with a line if not adjacent. AnkB/Actin or AnkB/Vinculin ratios are shown below. The position of the 220 kDa molecular weight marker from PageRuler Plus is indicated and coincides with the AnkB220-HA band.

Figure S9: Sema3F-Induced Spine Retraction is Impaired in Neuronal Cultures from *Ank2 ^+/-^* Mice Expressing the ASD Mutation AnkB A368G.

Raw images of *Ank2 ^+/-^* cortical neuronal cultures transfected with pCAG-IRES-EGFP and WT AnkB-220 or mutant AnkB-220 A368G are shown. Cells were treated on DIV14 with 5 nm Fc (control) or Sema3F-Fc for 30 min, and immunostained for EGFP. Representative confocal images of EGFP-labeled apical dendrites on pyramidal neurons are shown. Scale bar = 3 µm. The same scale is used for all panels.
