# Supplementary material for: Structural interactions of ankyrin B with NrCAM and β2 spectrin
Source: J Biol Chem. 2025 Oct 30;301(12):110872. doi: 10.1016/j.jbc.2025.110872 (PMC12681835; doi:10.1016/j.jbc.2025.110872)
Supplement: Supporting Table S1 [file mmc2.docx]

**Table S1: Consistency of interactions in the AnkB/NrCAM complex across five AlphaFold2 models**

| **Interacting Residue Pair** | **Interaction Type** | **Average Distance (Å)** | **Standard Deviation (Å)** | **No. of Models** |
| --- | --- | --- | --- | --- |
| ASP1269 (AnkB) - ARG308 (NrCAM) | H-bond | 2.91 | 0.32 | 3 |
| ASP1269 (AnkB) - GLN341 (NrCAM) | H-bond | 2.63 | 0.23 | 4 |
| GLN1265 (AnkB) - LYS275 (NrCAM) | H-bond | 2.5 | 0.27 | 4 |
| GLN1275 (AnkB) - LYS408 (NrCAM) | H-bond | 3.21 | 0.04 | 2 |
| GLU1268 (AnkB) - ASN331 (NrCAM) | H-bond | 3.24 | 0.09 | 5 |
| GLU1268 (AnkB) - THR329 (NrCAM) | H-bond | 2.16 | 0.19 | 4 |
| GLU1283 (AnkB) - ARG463 (NrCAM) | Salt Bridge | 2.36 | 0.14 | 5 |
| GLU1286 (AnkB) - ARG463 (NrCAM) | Salt Bridge | 3.35 | 0.22 | 2 |
| GLU1286 (AnkB) - ARG506 (NrCAM) | Salt Bridge | 3.51 | 0.18 | 2 |
| GLU1289 (AnkB) - ARG539 (NrCAM) | Salt Bridge | 2.82 | 0.05 | 2 |
| GLU1292 (AnkB) - LYS562 (NrCAM) | H-bond | 2.9 | 0.07 | 5 |
| GLY1274 (AnkB) - ASP364 (NrCAM) | H-bond | 2.81 | 0.08 | 4 |
| PRO1294 (AnkB) - TYR606 (NrCAM) | H-bond | 2.82 | 0.02 | 2 |
| SER1271 (AnkB) - HIS374 (NrCAM) | H-bond | 3.14 | 0.12 | 4 |
| SER1291 (AnkB) - LYS572 (NrCAM) | H-bond | 2.72 | 0.21 | 2 |
| SER1302 (AnkB) - LYS638 (NrCAM) | H-bond | 3.18 | 0.08 | 2 |
| TYR1258 (AnkB) - HIS242 (NrCAM) | H-bond | 2.64 | 0.29 | 3 |
| TYR1276 (AnkB) - HIS374 (NrCAM) | H-bond | 2.99 | 0.11 | 5 |
