# Supplementary material for: Structural interactions of ankyrin B with NrCAM and β2 spectrin
Source: J Biol Chem. 2025 Oct 30;301(12):110872. doi: 10.1016/j.jbc.2025.110872 (PMC12681835; doi:10.1016/j.jbc.2025.110872)
Supplement: Supporting Table S2 [file mmc3.docx]

**Table S2: Residue interaction characteristics for AnkB/NrCAM complex**

| Interacting Residue Pair | Interaction Type | Distance (Å) |
| --- | --- | --- |
| ARG40 (AnkB) - ALA1206 (NrCAM) | H-bond | 2.28 |
| LYS73 (AnkB) - GLU1204 (NrCAM) | H-bond | 2.83 |
| LYS96 (AnkB) - GLU1202 (NrCAM) | H-bond | 1.99 |
| ASN98 (AnkB) - TYR1198 (NrCAM) | H-bond | 2.29 |
| TYR135 (AnkB) - GLY1196 (NrCAM) | H-bond | 3.10 |
| GLN139 (AnkB) - TYR1198 (NrCAM) | H-bond | 2.79 |
| GLN139 (AnkB) - TYR1198 (NrCAM) | H-bond | 2.85 |
| GLN173 (AnkB) - TYR1225 (NrCAM) | H-bond | 2.10 |
| ARG201 (AnkB) - SER1254 (NrCAM) | H-bond | 3.01 |
| SER232 (AnkB) - TYR1258 (NrCAM) | H-bond | 2.66 |
| ILE239 (AnkB) - TYR1258 (NrCAM) | H-bond | 2.79 |
| ASP298 (AnkB) - GLU1268 (NrCAM) | H-bond | 2.94 |
| ARG308 (AnkB) - ASN1267 (NrCAM) | H-bond | 2.20 |
| ASN331 (AnkB) - GLU1268 (NrCAM) | H-bond | 3.31 |
| ASP364 (AnkB) - GLY1274 (NrCAM) | H-bond | 3.04 |
| HIS374 (AnkB) - TYR1276 (NrCAM) | H-bond | 2.25 |
| HIS374 (AnkB) - TYR1276 (NrCAM) | H-bond | 2.25 |
| LYS562 (AnkB) - GLU1289 (NrCAM) | H-bond | 3.21 |
| ARG37 (AnkB) - ASP1209 (NrCAM) | Salt Bridge | 1.78 |
| ARG40 (AnkB) - ASP1205 (NrCAM) | Salt Bridge | 3.57 |
| LYS73 (AnkB) - ASP1205 (NrCAM) | Salt Bridge | 3.79 |
| LYS96 (AnkB) - GLU1202 (NrCAM) | Salt Bridge | 1.99 |
| ARG201 (AnkB) - ASP1252 (NrCAM) | Salt Bridge | 3.92 |
| ARG463 (AnkB) - GLU1283 (NrCAM) | Salt Bridge | 3.39 |
| ARG495 (AnkB) - GLU1286 (NrCAM) | Salt Bridge | 2.97 |
| LYS562 (AnkB) - GLU1289 (NrCAM) | Salt Bridge | 3.21 |
