# Supplementary material for: Structural interactions of ankyrin B with NrCAM and β2 spectrin
Source: J Biol Chem. 2025 Oct 30;301(12):110872. doi: 10.1016/j.jbc.2025.110872 (PMC12681835; doi:10.1016/j.jbc.2025.110872)
Supplement: Supporting Table S3 [file mmc4.docx]

**Table S3: Consistency of interactions in the AnkB/β2-Spectrin complex across five AlphaFold models**

| **Interacting Residue Pair** | **Interaction Type** | **Average Distance (Å)** | **Standard Deviation (Å)** | **No. of Models** |
| --- | --- | --- | --- | --- |
| SER971 (AnkB) - THR1796 (β2-Spectrin) | H-bond | 2.83 | 0.2 | 5 |
| ARG1003 (AnkB) - ALA1721 (β2-Spectrin) | H-bond | 2.69 | 0.02 | 5 |
| ARG1003 (AnkB) - SER1723 (β2-Spectrin) | H-bond | 2.83 | 0.1 | 5 |
| THR999 (AnkB) - ALA1875 (β2-Spectrin) | H-bond | 2.85 | 0.01 | 5 |
| ARG985 (AnkB) - ASP1789 (β2-Spectrin) | Salt Bridge | 2.8 | 0.39 | 5 |
| ARG985 (AnkB) - GLU1785 (β2-Spectrin) | H-bond | 2.58 | 0.03 | 5 |
| LEU969 (AnkB) - GLU1792 (β2-Spectrin) | H-bond | 2.68 | 0.07 | 4 |
| VAL970 (AnkB) - GLU1792 (β2-Spectrin) | H-bond | 2.28 | 0.07 | 4 |
| ARG1007 (AnkB) - ASP1789 (β2-Spectrin) | H-bond | 3.1 | 0.3 | 3 |
| ARG1007 (AnkB) - GLU1718 (β2-Spectrin) | H-bond | 3.02 | 0.19 | 5 |
| ARG1062 (AnkB) - GLU1785 (β2-Spectrin) | Salt Bridge | 2.92 | 1.07 | 4 |
