# Supplementary material for: Structural interactions of ankyrin B with NrCAM and β2 spectrin
Source: J Biol Chem. 2025 Oct 30;301(12):110872. doi: 10.1016/j.jbc.2025.110872 (PMC12681835; doi:10.1016/j.jbc.2025.110872)
Supplement: Supporting Table S4 [file mmc5.docx]

**Table S4: Residue interaction characteristics for AnkB/β2-Spectrin complex**

| Interacting Residue Pair | Interaction Type | Distance (Å) |
| --- | --- | --- |
| ARG1003 (AnkB) - ALA1721 (β2-Spectrin) | H-bond | 2.70 |
| ARG1003 (AnkB) - SER1723 (β2-Spectrin) | H-bond | 2.98 |
| ARG985 (AnkB) - GLU1785 (β2-Spectrin) | H-bond | 2.54 |
| VAL970 (AnkB) - GLU1792 (β2-Spectrin) | H-bond | 2.25 |
| SER971 (AnkB) - GLU1792 (β2-Spectrin) | H-bond | 2.65 |
| LEU969 (AnkB) - GLU1792 (β2-Spectrin) | H-bond | 2.57 |
| SER971 (AnkB) - THR1796 (β2-Spectrin) | H-bond | 2.94 |
| ALA976 (AnkB) - ALA1873 (β2-Spectrin) | H-bond | 3.20 |
| THR999 (AnkB) - ALA1875 (β2-Spectrin) | H-bond | 2.84 |
| ARG985 (AnkB) - GLU1785 (β2-Spectrin) | Salt Bridge | 3.89 |
| ARG985 (AnkB) - ASP1789 (β2-Spectrin) | Salt Bridge | 2.96 |
