# Supplementary material for: Structural interactions of ankyrin B with NrCAM and β2 spectrin
Source: J Biol Chem. 2025 Oct 30;301(12):110872. doi: 10.1016/j.jbc.2025.110872 (PMC12681835; doi:10.1016/j.jbc.2025.110872)
Supplement: Supporting Table S5 [file mmc6.docx]

**Table S5. Characteristics of ASD-Associated Variants in AnkB**

| Variant | Inheritance | AlphaMissense Score (Classification) | Allele Frequency (gnomAD) | HGMD Association |
| --- | --- | --- | --- | --- |
| A368G | Maternally Inherited | 0.706 (Pathogenic) | Not observed | ASD |
| A373V | Maternally Inherited | 0.791 (Pathogenic) | 2.29×10^-5^ | Not Reported^1^ |
| A525V | Maternally Inherited | 0.402 (Benign) | 4.34×10^-6^ | ASD |
| E819K | Maternally Inherited | 0.593 (Ambiguous) | 6.2×10^-7^ | ASD |
| R977Q | Maternally Inherited | 0.977 (Pathogenic) | 2.42×10^-5^ | Not Reported^1^ |
| I807M | De Novo | 0.852 (Pathogenic) | Not Observed | ASD |
| P1380R | De Novo | 0.955 (Pathogenic) | Not Observed | ASD |

**Notes**: ^1^ ASD-related variants reported in (Wang, T, *et al*., *Nat Commun* 11(2016) 1-13).

^2^AlphaMissense scores ≥0.7 are classified as pathogenic, <0.7 as benign or ambiguous (based on proximity to threshold). Allele frequencies are from gnomAD v4.1.0; *de novo* variants are not observed in population databases. HGMD associations are specific to ASD, with no reported links to intellectual disability, bipolar disorder, or seizures (Stenson, P.D. *et al*., *Hum Genet* 139 (2020) 1197-1207).
